# The effect of bisphosphonates on the endothelial differentiation of mesenchymal stem cells

**DOI:** 10.1038/srep20580

**Published:** 2016-02-09

**Authors:** Dileep Sharma, Stephen Mark Hamlet, Eugen Bogdan Petcu, Saso Ivanovski

**Affiliations:** 1 Molecular Basis of Disease Group, Regenerative Medicine Centre, Menzies Health Institute, QLD, School of Dentistry and Oral Health, Griffith University, Gold Coast Campus, Australia; 2 Molecular Basis of Disease Group, Regenerative Medicine Centre, Menzies Health Institute, QLD, School of Medicine, Griffith University, Gold Coast Campus, Australia

## Abstract

The contribution of the local stem cell niche to providing an adequate vascular framework during healing cannot be overemphasized. Bisphosphonates (BPs) are known to have a direct effect on the local vasculature, but their effect on progenitor cell differentiation is unknown. This *in vitro* study evaluated the effect(s) of various BPs on the differentiation of human placental mesenchymal stem cells (pMSCs) along the endothelial lineage and their subsequent functional and morphogenic capabilities. pMSC multipotency was confirmed by successful differentiation into cells of both the osteogenic and endothelial lineages, as demonstrated by positive Alizarin Red S staining and Ac-LDL uptake. pMSC differentiation in the presence of non-cytotoxic BP concentrations showed that nitrogen containing BPs had a significant inhibitory effect on cell migration and endothelial marker gene expression, as well as compromised endothelial differentiation as demonstrated using von Willebrand factor immunofluorescence staining and tube formation assay. This *in vitro* study demonstrated that at non-cytotoxic levels, nitrogen-containing BPs inhibit differentiation of pMSCs into cells of an endothelial lineage and affect the downstream functional capability of these cells supporting a multi-modal effect of BPs on angiogenesis as pathogenic mechanism contributing to bone healing disorders such as bisphosphonate related osteonecrosis of the jaws (BRONJ).

Bone is a dynamic tissue constantly remodelled by the sequential removal of mature tissue by osteoclasts and its replacement through the deposition of newly formed mineralized matrix by osteoblasts^1^. The local vasculature within this bone multicellular unit (BMU) has a crucial role in modulating the bone formation and resorption processes^2^. Furthermore, the local vasculature provides an important niche of multipotent (stem) cells that can differentiate into bone forming cells and newly formed blood vessels^3^.

Bisphosphonates are a group of drugs that have a structural similarity to pyrophosphate and a high affinity for mineralised tissue, making them suitable agents for inhibiting osteoclastic bone resorption^4^. There are two main classes of BPs that differ in potency and mode of action, namely the low potency, non-nitrogen containing BPs including clodronate (CLO) and Etidronate, and the more commonly used higher potency, nitrogen containing BPs including Alendronate (ALN), Ibandronate and Zoledronate (ZA)^5^,^6^. Bisphosphonates have been widely used for their multimodal bone-sparing action and to prevent the development of osteolytic lesions in various cancers^7^,^8^. Recent studies suggest that bisphosphonates could have an antitumor action through their effect on the local vasculature^4^,^5^.

BPs have also been associated with the pathogenesis of a locally destructive oral condition called BP-Related Osteonecrosis of the Jaws (BRONJ)^9^,^10^. BRONJ is mainly associated with the use of high potency intravenous ZA, less commonly with orally administered ALN and rarely with less potent BPs^11^. Putative risk factors for BRONJ include the use of high potency BPs along with invasive surgical procedures in the oral and perioral area, infections and trauma to the jaw bones, as well as concomitant exposure to immunosuppressive and/or chemotherapy drugs^6^,^12^,^13^. Various hypotheses have been proposed to explain the etiopathogenic mechanisms of BRONJ, including the notion that the inhibitory effects of bisphosphonates on osteoclasts can leads to impaired bone remodelling, and the possibility of a toxic effect on oral epithelial keratinocytes^14^,^15^. More recent reports suggest that BPs could have an anti-angiogenic role leading to a state of local chronic ischemia that could contribute significantly to the pathogenesis of BRONJ^16^,^17^.

It is widely accepted that local angiogenesis is an essential part of bone healing and hence impairment by BPs would negatively influence both bone formation and homeostatic remodelling. It is not known if the anti-angiogenic role of BPs is exerted at the level of precursor stem cells or on mature blood vessels. This *in vitro* study aims to evaluate the effects of nitrogen and non-nitrogen containing BPs on the endothelial differentiation potential of human term placental mesenchymal stem cells (pMSCs) in order to assess whether a perturbation in stem cell differentiation by BPs could play a role in the pathophysiology of bone healing disorders such as BRONJ.

## Results

### Stem cell proliferation and viability

The first experiment aimed at evaluating the direct effect of various BPs on the pMSCs *in vitro*. pMSCs were incubated in 100 μl aliquots of endothelial growth media (EGM-2) with 0.25–200 μM clodronate, alendronate and zoledronate (Sigma-Aldrich, Castle Hill, NSW, Australia) for 10 days to determine the non-cytotoxic doses of BPs to be utilized for subsequent differentiation experiments by alamarBlue^^®^^ and Live/Dead^®^ assays over multiple time points.

**Figure 1:**
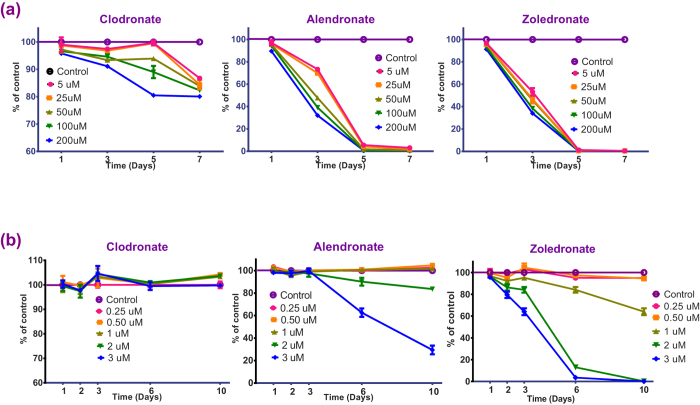
(**a,b**) The effect of bisphosphonates on pMSC proliferation. 2 μM CLO, 1 μM ALN and 0.5 μM ZA were considered to be optimal concentrations for the differentiation studies without adversely affecting cell survival over a 10-day period.

High bisphosphonate concentrations (>5 μM) had a significant negative effect on pMSC proliferation (Fig. 1). After one day of treatment, only CLO could be tolerated at all the concentrations tested >5 μM. However, by day 3 all of the samples that were exposed to CLO concentrations beyond 25 μM showed a statistically significant reduction in cell proliferation. By the end of 5 and 7 days of exposure, all the tested CLO concentrations showed a significant suppression of cell growth. In contrast, exposure to high (>5 μM) ALN and ZA concentrations negatively affected pMSC proliferation from the earliest (day 1) time point and this inhibition of cell proliferation was observed to increase even further over the experimental period (Fig. 1a).

At lower BP concentrations (0.25, 0.5, 1, 2 & 3 μM), the addition of CLO to the cell culture media did not have any significant effect on proliferation over the experimental time period of 10 days. ALN concentrations ≤1 μM and ZA concentrations ≤0.5 μM did not affect pMSC survival over the 10 day duration of exposure (Fig. 1b). Based on these observations, 2 μM CLO, 1 μM ALN and 0.5 μM ZA were considered to be the maximal concentrations that could be used for the differentiation studies without adversely affecting cell proliferation over a 10 day period.

**Figure 2:**
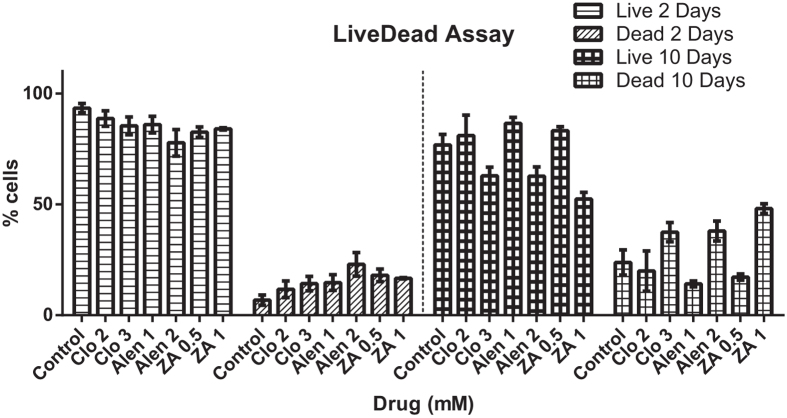
Live/Dead^^®^^ analysis of pMSCs cultured in BP containing media. At the optimal BP concentrations: CLO 2 μM, ALN 1 μM and ZA 0.5 μM, as suggested by the cell proliferation study (Fig. 1), significantly more live cells (and fewer dead cells) were observed after 10 days of culture.

To confirm the absence of any cytotoxic effects at the selected drug concentrations, a Live/Dead^®^ assay was performed after pMSCs were exposed to CLO (2 μM & 3 μM), ALN (1 μM & 2 μM), and ZA (0.5 μM & 1 μM) for 10 days. After 2 days incubation, while the numbers of live cells were lower in cultures exposed to the higher potency ALN and ZA compared to the control untreated cells, there was no observable significant concentration dependent difference in pMSC viability. By day 10 of culture however, significantly more live cells (and fewer dead cells) were observed in the cultures with the lower of the two BP concentrations tested (Fig. 2). These results confirm that the lower concentrations (of the two tested) were not cytotoxic at both the early and late time points and were suitable for the differentiation studies (Fig. 2).

### Scratch wound healing assay

**Figure 3:**
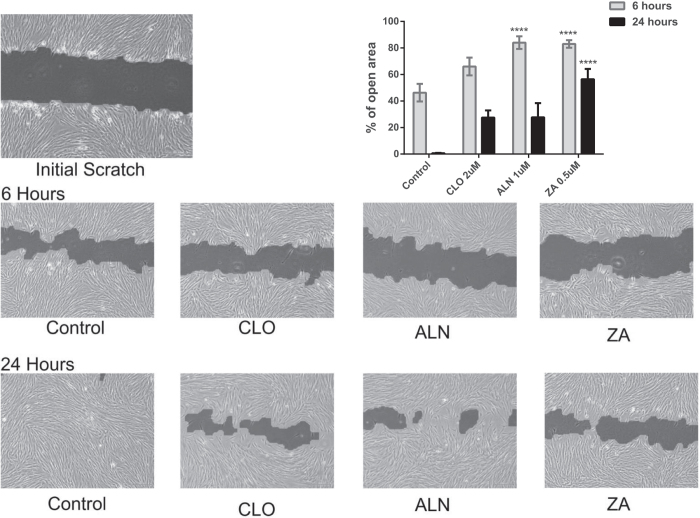
Light microscope images show *in vitro* wound healing after 6 hrs and 24 hrs was significantly inhibited in the presence of BPs. Furthermore, quantitative analysis (histogram) showed that inhibition of wound healing by the higher potency ALN (at 6 hrs) and ZA (at both 6 and 24 hours) were both significantly higher (p < 0.0001) than with the lower potency CLO.

The effect of non-cytotoxic levels of BPs (derived from above experiments) on pMSCs migration was assessed and quantified using a scratch wound healing assay. pMSCs were seeded on a 24-well plate and grown to 100% confluence in standard growth media. A scratch across the base of each well was made and cells were then incubated in media of either EGM-2 alone or EGM-2 with 2 μM CLO, 1 μM ALN or 0.5 μM ZA, for 6 and 24 hours. The open wound area was measured at each time point and compared with that of corresponding controls (Fig. 3). The open wound area was significantly higher with all BP drugs tested at both time points. However, in the presence of ALN (at 6 hrs) and ZA (at both 6 and 24 hours) the difference in open wound areas were significantly higher (p < 0.0001) than CLO (Fig. 3).

### pMSC Osteogenic Differentiation

**Figure 4:**
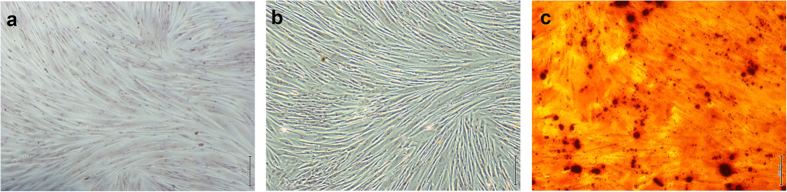
Osteogenic differentiation capability of (**a**) pMSCs. Following culture in osteogenic media for 2 weeks (**b**), the cells showed significant Alizarin Red S staining of a mineralised cell matrix (**c**).

To study the effect of BPs on endothelial differentiation, the cells needed to be tested for multipotency and their ability to differentiate. The pMSCs were seeded into 24-well microtitre plates and cultured in standard media supplemented with 10 mM β-glycerophosphate, 100 nM dexamethasone, and 0.2 mM ascorbic acid (Osteogenic media). After two weeks of culture in osteogenic media, Alizarin Red S staining of the pMSCs showed positive Alizarin red staining confirming the production of mineralized matrix by the stem cells (Fig. 4).

### pMSC endothelial differentiation and Dil-Ac-LDL labelling

**Figure 5:**
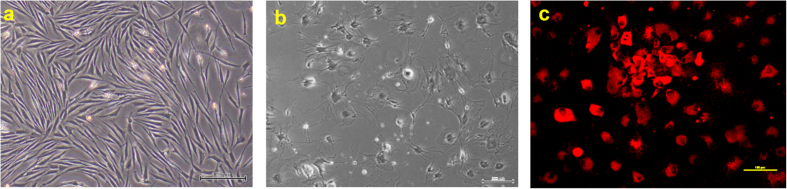
Endothelial lineage of the VEGF treated pMSCs was confirmed by Ac-LDL uptake after 10 days. (**a**) Control pMSCs, (**b**) Endothelial differentiated pMSCs and (**c**) Endothelial differentiated pMSCs showing uptake of fluorescent labelled Ac-LDL.

To induce endothelial differentiation, the pMSCs were cultured in EGMV media (EGM-2 supplemented with 50 ng/ml VEGF). Following 10 days of differentiation in EGMV, there was a distinct difference in the morphology of the pMSCs evident on bright field microscopy. The cells now appeared more stellate with multiple cell projections compared to spindle shaped cells in the case of the untreated controls (Fig. 5a,b). An LDL uptake assay that specifically labels endothelial cells, confirmed that a significant proportion of pMSCs differentiated into endothelial-like cells (Fig. 5c).

### Immunostaining for Von Willebrand factor (vWF)

**Figure 6:**
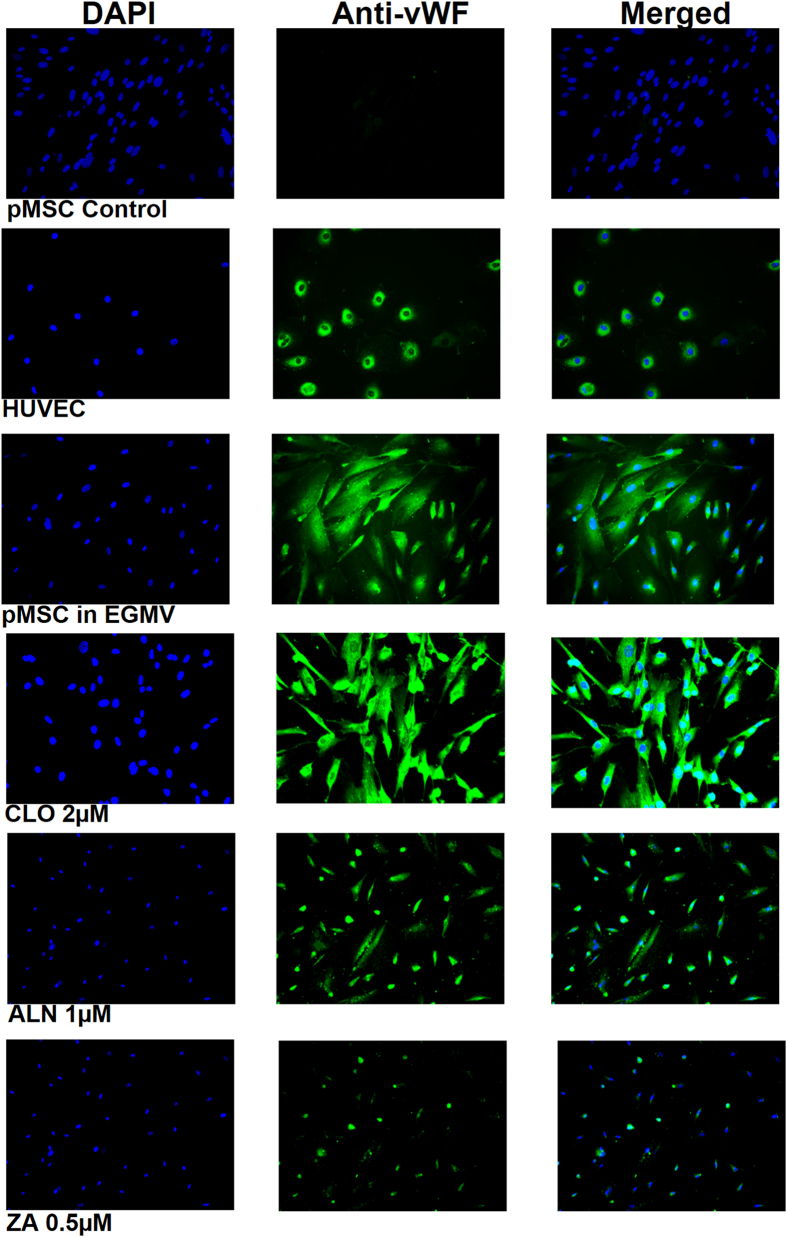
pMSCs differentiation into endothelial cells as assessed by the intensity of Von Willebrand factor staining was inhibited by both ALN and ZA. Compared to unstimulated pMSCs, CLO had a minimal effect on vWF expression. vWF staining in HUVECs was included as a positive control. Legend: DAPI (4′,6-Diamidino-2-Phenylindole); cell nuclear stain, Anti-vWF; FITC labelled Von Willebrand factor, Merged; combined DAPI and anti-vWF staining, HUVEC; human umbilical vein endothelial cells, EGMV; endothelial growth media supplemented with VEGF

Von Willebrand factor (vWF) staining was performed at the end of ten days exposure of pMSCs to EGMV with or without BPs (CLO 2 μM, ALN 1 μM or ZA 0.5 μM) to evaluate the degree of differentiation qualitatively. vWF staining of HUVEC’s was included as a positive control. CLO had a minimal effect on the degree and intensity of vWF staining in the pMSCs compared to endothelial media alone. However, there was a significant inhibitory effect of both ALN and ZA on the intensity and degree of staining of pMSCs with the highest effect noted in response to ZA 0.5 μM (Fig. 6).

### Angiogenesis (tube formation) Assay

**Figure 7:**
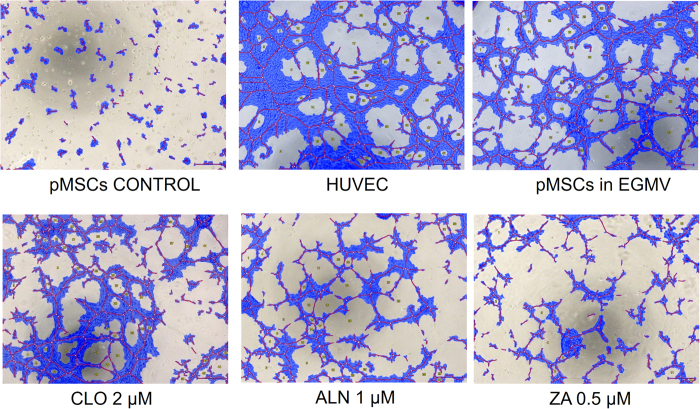
Wimasis WimTube (Wimasis GmbH Munich, Germany) generated image showing significantly reduced tube formation by pMSC derived endothelial cells in the presence of BPs compared to both stimulated pMSCs and HUVECs.

**Figure 8:**
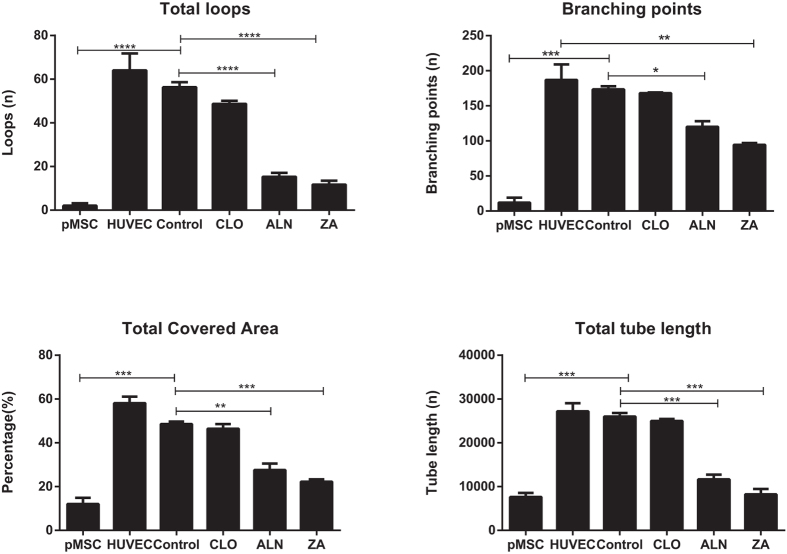
Quantitative analysis of the morphological tube formation parameters; total covered area, total loop length, loop numbers and the number of branching points (Wimasis WimTube, Wimasis GmbH Munich, Germany) were all significantly reduced in the pMSC derived endothelial cells in the presence of BPs compared to both stimulated pMSCs and HUVECs. Inhibition was potency dependent with the most significant inhibition seen in the ALN and ZA treated cells. (Significant differences, compared to EGMV are indicated as *p < 0.05, **p < 0.01, ***p < 0.001 and ****p < 0.0001).

A tube formation assay was used to assess the morphological capacity of the endothelial-like cells following differentiation. Again, HUVECs were used as a positive control. 50 μl cell suspension containing either 2 × 10^5^ pMSCs/ml in EGMV media with or without BPs (0.5 μM ZA, 1 μM ALN, 2 μM CLO) or HUVEC’s in EGMV were added and tube formation assessed and imaged at the end of 6 hrs. Commercial software service was used to quantify various tube formation parameters compared to controls (Figs 7 and 8).

The total loop lengths were significantly reduced (p < 0.0001) from 25,977 ± 1169 μm in the no drug control, to 11,660 ± 1475 μm and 8221 ± 1718 μm with ALN and ZA respectively. Similar significant trends were observed in the case of loop numbers (control 56.33 ± 4.04, ALN 15.33 ± 3.05 & ZA 11.67 ± 3.21, p < 0.0001), branching points (control 173.50 ± 6.36, ALN 120 ± 11.31 & ZA 94.50 ± 3.53, p = 0.03 & p = 0.0056) and total covered area (control 48.60 ± 1.55%, ALN 27.55 ± 4.17% & ZA 22.25 ± 1.49%, p = 0.0027 & p = 0.0008) (Fig. 8).

### Gene expression

The gene expression of stem cell (CD73 and CD105) and endothelial cell markers (PECAM-1, VEGFR1, VEGFR2, von Willebrand Factor and CD34) in pMSCs after 1, 5 and 10 days exposure to CLO (2 μM), ALN (1 μM) and ZA (0.5 μM) was evaluated in order to assess the effect of these drugs on the differentiation process.

**Figure 9:**
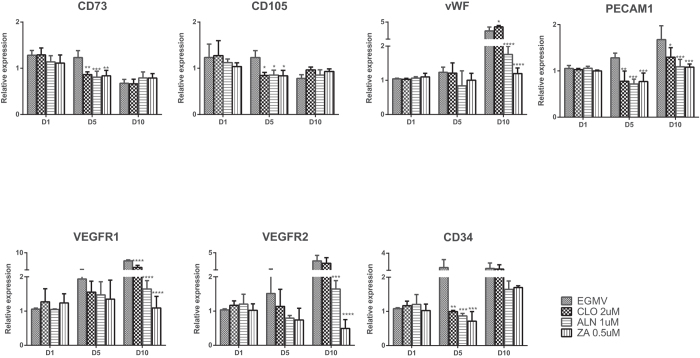
Relative gene expression of stem (CD73 and CD105) and endothelial (von Willebrand Factor, PECAM-1, VEGF-R1, VEGF-R2, and CD34) cell markers in pMSCs after 1, 5 and 10 days differentiation in EGMV alone or with the BPs CLO (2 μM), ALN (1 μM) or ZA (0.5 μM). Significant differences in expression compared to EGMV are indicated as *p < 0.05, **p < 0.01, ***p < 0.005 and ****p < 0.0001.

The expression of both CD73 and CD105 were down-regulated over time in pMSCs cultured in endothelial differentiation media (EGMV) alone, or supplemented with BPs (Fig. 9). Although the loss in stem cell marker expression was significantly higher in the BP treated cells at day 5, by the end of the observation period there were no significant differences in the levels of CD 73 and CD 105 expression between the BP treated and untreated pMSCs. These results are commensurate with a loss of “stemness” suggesting successful differentiation into endothelial phenotype by the end of the experimental period. The shift in pMSCs to an endothelial phenotype was confirmed by the increased expression over culture time of the endothelial markers: von Willebrand Factor, PECAM-1, VEGFR1, VEGFR2, and CD34 (Fig. 9).

In the absence of BPs, vWF expression by the endothelial growth media stimulated pMSCs showed an increasing trend over the experimental period with a significant 4-fold up-regulation (p < 0.0001) in expression at the end of ten days. The presence of BPs (CLO, ALN, ZA) in the culture media had a minimal effect at the end of five days, however by ten days both ALN and ZA showed a significant inhibitory effect (p < 0.0001) on vWF expression (1.5–2 fold increase) compared to the EGM stimulated control (Fig. 9).

A similar trend in PECAM-1 expression was observed wherein differentiation of pMSCs in EGMV led to a significant increase (p = 0.0023) in expression at the end of the experimental period. However, a statistically significant inhibitory effect by all of the BPs tested was evident at both the early (five days) and late (ten days) time points in comparison to the EGMV stimulated control (Fig. 9).

VEGF-R1 and VEGF-R2 expression by the pMSCs showed the highest fold change increases (~9-fold and 5-fold respectively) with differentiation in EGMV media alone over the experimental period (Fig. 9). In the presence of all three bisphosphonates, VEGF-R1 expression was lower than the EGM control by five days and significantly lower by ten days (p < 0.0001). Similarly, ALN and ZA also significantly inhibited VEGF-R2 expression after ten days (p = 0.0002 and p < 0.0001 respectively).

CD34 expression in untreated cells showed a maximal three-fold increase in expression by five days (p = 0.0489). Whilst BP exposure significantly reduced CD34 expression (CLO, p = 0.0154; ALN, p = 0.006; ZA, p = 0.0019) in the short-term (five day time point), this effect was not maintained over the 10 day period of EGM stimulation. (Fig. 9).

In summary, the presence of BPs during endothelial differentiation of pMSCs significantly inhibited the expression of the endothelial markers with highest effects observed on exposure to ZA (Fig. 9).

## Discussion

This study presents novel evidence that the documented anti-angiogenic effects of BPs may also involve inhibition of progenitor stem cell differentiation into endothelial cells, a role beyond any direct cytotoxic effect of BPs on vascular cells. Pluripotent stem cells present locally and in the circulation have been shown to play a significant role in wound healing and their differentiation into the cell types essential for tissue repair and regeneration is an integral part of tissue homeostasis^18^,^19^. Various sources and protocols to isolate stem cells have been well documented in the literature and allogenic umbilical cord derived stem cells have been a focus of research as they can be obtained with ease and in large quantities^20^,^21^. Further, pMSCs are well documented to possess the ability to differentiate into osteoblasts, adipose cells, chondroblasts and neural cells, when exposed to appropriate stimuli and culture conditions^22^,^23^,^24^,^25^. Similarly, we have also demonstrated that the pMSCs used in the present study were able to differentiate into both osteogenic and endothelial phenotypes. The process of differentiation of stem cells along the endothelial lineage has attracted considerable interest in recent times^26^,^27^. However, there are no reported studies evaluating the effect of BPs on pluripotent cells and their downstream differentiation.

*In vitro* studies have documented the inhibitory effect of BPs on a variety of mature vascular cells including HUVECs, EPC and ECFCs^28^,^29^. The results of this study are in keeping with those by Ribatti et al (2008) that demonstrate a significant reduction in cell growth in a dose dependent manner beyond 10 μM of CLO exposure. Also, CLO was observed to significantly affect morphogenesis (tube formation) and angiogenesis as quantified by a chorioallantoic membrane (CAM)-gelatin sponge assay in the presence of FGF-2, a known angiogenic cytokine. Similar findings were reported by Walter et al (2011) who evaluated the effects of CLO, Ibandronate, Pamidronate and ZA at concentrations ranging from 5 to 500 μM. HUVEC viability was affected by CLO at 200 μM and all of the other BPs had a significant effect above 50 μM. Further, TUNEL and migration assays showed that the drugs affected various functions of HUVECs, as well as osteoblasts and fibroblasts. The results reported in the present study follow a similar pattern but vary in the magnitude of the reported effect. The BP concentrations used are also much lower than the studies involving HUVECs and other cell types suggesting that undifferentiated cells may be more susceptible to the inhibitory effect of BPs.

Cell migration is a vital component in healing and BPs, in particular the nitrogen containing ALN and ZA, are known to affect the migration of HUVEC cells *in vitro*^29^. This was also observed in our study wherein all BPs inhibited the migration of pMSCs in endothelial media. This inhibition was most significant in the case of ALN and ZA, which can be attributed to their higher potency.

The gene expression profile of pMSC’s exposed to endothelial differentiation media and BPs has not been previously documented. In this paper, pMSC exposure to BPs during endothelial differentiation was shown to have a detrimental effect, as evidenced by the continued expression of stem cell markers alongside statistically significant down-regulation of endothelial cell markers. Further, it was evident that ZA had the most significant effect whereas CLO with its lower potency showed comparable effects to the control (EGMV stimulated) cells. These gene expression results were corroborated by the immunocytochemical staining (vWF) results and the functional tube formation assays.

The direct effects of BPs on circulating vascular cells have been reported in a few *in vivo* studies. In clinical studies, a sustained reduction in circulating endothelial cells and their precursors was reported following repeated and intermittent ZA administration in early prostate cancer patients^30^,^31^,^32^. Furthermore, an increase in endothelial cell apoptosis in BRONJ patients, as well as multiple myeloma patients after BP administration, has also been reported^33^,^34^. However, one study reported that local vascularization was not affected in BRONJ-related mucoperiosteal tissues, suggesting that new blood vessel formation, rather than the existing vasculature, may be affected during the pathogenesis of BRONJ^35^. Taken together with the existing literature, the present study suggests that BPs can exert an inhibitory effect on the local stem cell populations and their functional role in replenishing the endothelial cell population, thus negatively affecting the early phases of bone healing.

This study also found that the high potency nitrogen-containing ZA, and to a lesser extent ALN, had a significantly higher effect than CLO on the differentiation process by diminishing the morphogenic capability of the differentiated stem cells. This is consistent with the clinical observation that BRONJ is more often associated with ZA than ALN use. This could be due to the fact that clinically administered BPs, owing to their high affinity to mineralised tissues, are immediately bound to the mineralised tissue limiting the effect of the BPs on the progenitor cell population^6^. However, on initiation of active bone remodelling they are released from the bone to exert their intended action of inhibiting the process of bone resorption. The BPs thus released during homeostatic remodelling may in fact negatively influence the local vascular compartment through their effect on differentiating progenitor cells. This however needs to be confirmed by additional *in vivo* experiments designed to evaluate the effect of BPs on local progenitor stem cell populations.

The present study suggests a novel mechanism in the pathogenesis of BRONJ whereby high-potency nitrogen-containing bisphosphonates have an anti-angiogenic effect by disrupting the differentiation of endothelial progenitor cells. *In vivo* studies are needed to verify this proposed pathogenic mechanism of bisphosphonates that may impair angiogenesis resulting in a BRONJ lesion. It would also be important to determine if this action is exerted in both the bone tissues and the adjacent mucosal regions.

## Methods

### Cell Culture

#### Stem Cells

Human term placental mesenchymal stem cells (pMSCs), positive for the MSC markers CD29, CD44, CD73, CD90 and CD105 but negative for the endothelial progenitor markers CD31, CD34 and CD45 were sourced from the laboratory of Prof K. Khosrotehrani^36^. These pMSCs have been shown to be able to differentiate down both osteogenic and adipogenic lineages confirming their MSC status, and their *in vivo* angiogenic capacity has been recently demonstrated^36^,^37^,^38^. The pMSCs were expanded in DMEM supplemented with 10% Fetal Calf Serum and 1% Pencillin-Streptomycin mixture and incubated in a 5% CO_2_ atmosphere at 37 °C. Cells from passage 2 to 6 were utilised.

### Cell growth and viability

#### Cell growth

pMSCs (100 μl @ 2 × 10^4^ cells/ml) were plated onto a 96 well microtitre-plate and allowed to attach for 24 hrs. The cells were subsequently incubated in 100 μl aliquots of endothelial growth media (EGM-2, Lonza, Mount Waverley VIC, Australia) with 0.25–200 μM clodronate, alendronate and zoledronate (Sigma-Aldrich, castle Hill, NSW, Australia) for 10 days. The proportion of viable cells and the proliferation rate compared to untreated (control) cells was determined by alamarBlue^®^ assay (Life Technologies, Mulgrave, VIC, Australia).

#### Cell viability

pMSCs (100 μl @ 2 × 10^5^ cells/ml) were plated into a 96 well microtitre-plate and cultured to 70% confluence. EGM-2 with or without BPs (2 and 3 μM CLO, 1 and 2 μM ALN and 0.5 and 1 μM ZA) was added and the cells incubated at 37 °C and 5% CO_2_. A LIVE/DEAD^^®^^ assay (Life Technologies, Mulgrave, VIC, Australia) was performed after 48 hours and 10 days of culture. Briefly, the cells were washed 3 times with PBS prior to the assay. A working solution of 1 μM Calcein AM and 2 μM EthD-1 in sterile PBS was added to each well (100 ul/well) and fluorescence visualised with an epi-fluorescence microscope. The relative percentages of live and dead cells were quantified spectro-fluorometrically.

### Cell migration assay

pMSCs were seeded on a 24-well plate at 2 × 10^4^ cells/well and grown to 100% confluence in standard growth media (DMEM supplemented with 10% FCS, 1% penicillin-streptomycin and 1% non-essential amino acids) before incubating overnight in a serum-reduced standard medium (1% FCS). A scratch across the base of each well was made with a 200 μL sterile pipette tip and the wells washed extensively with PBS to remove the detached cells and debris. The cells were then incubated in a test media (EGM-2 alone or EGM-2 with 2 μM CLO, 1 μM ALN or 0.5 μM ZA) for 6 and 24 hours when the scratches were imaged at 4X magnification. The images were analysed and the percentage of open area quantified using automated image analysis with the TScratch software (CSElab, Zurich, Switzerland)^39^.

### Osteogenic differentiation

To confirm the osteogenic potential of the pMSCs, 2 × 10^4^ cells/well were seeded into 24-well microtitre plates and cultured in standard media supplemented with 10 mM β-glycerophosphate, 100 nM dexamethasone, and 0.2 mM ascorbic acid (Sigma, Castle Hill, NSW, Australia). This osteogenic medium was refreshed twice weekly and at the end of two weeks, Alizarin Red S staining was used to assess the calcium content of the cells in culture. The staining was carried out using a modification of the method previously described^15^. Briefly, the cell monolayer was first washed in PBS then fixed using 10% formalin for 10 minutes. The cells were then washed with PBS and 1 ml of 2% Alizarin red S was added for 20 minutes. Subsequently the wells were washed four times with PBS and staining was assessed visually.

### Endothelial differentiation

Confluent pMSCs were passaged into T-25 flasks containing EGMV media (EGM-2 supplemented with 50 ng/ml VEGF, ISOkine^™^, ORF Genetics, Iceland), and cultured for ten days. Media was refreshed every three days and the cells tested for endothelial differentiation using an LDL uptake assay (described below) at the end of ten days.

#### DIL-AC-LDL Assay

The uptake of Dil-Ac-LDL by the pMSCs following differentiation in EGMV media was used to assess their resultant endothelial cell phenotype^40^. Ac-LDL, labelled with the fluorescent probe 1,1′-dioctadecyl-3,3,3′,3′-tetramethyl-indocarbocyanine perchlorate (Dil-Ac-LDL, Alfa Aesar, Lancashire, United Kingdom) was used as per the manufacturer’s instructions. Briefly, the endothelial-like cells were pre-incubated in serum free medium containing 1% BSA for 48 hours. Subsequently, cells were incubated with 10 micrograms/ml Dil-Ac-LDL for 5 hrs at 37 °C in a 5% CO_2_ atmosphere and subsequently examined by fluorescence microscopy.

### BPs exposure and endothelial differentiation

pMSCs were cultured in EGMV media with BPs (2 μM CLO, 1 μM ALN and 0.5 μM ZA) for ten days in T25 flasks. To evaluate and quantify the effect of BPs on endothelial differentiation, immunocytochemistry and angiogenesis assay were performed at the end of 10 days with media refreshed every three day. Human umbilical vein endothelial cells (HUVEC, Lonza, Mount Waverley VIC, Australia) cultured in EGMV media served as a positive control for these assays.

#### Immunocytochemistry

Von Willebrand factor (vWF) staining was performed at the end of ten days exposure of pMSCs to EGMV with or without BPs (CLO 2 μM, ALN 1 μM or ZA 0.5 μM). The detached cells were grown onto poly-L-lysine coated coverslips and stained as per the manufacturer’s instructions. Briefly, cells were fixed at room temperature with chilled methanol (−20 °C) for 5 minutes and washed twice with ice cold PBS. After permeabilization for 10 min with PBS containing 0.2% Triton X-100 and blocking with 1% BSA in PBS, anti-vWF antibody (Abcam, Melbourne, VIC, Australia) was added onto the cells and incubated at room temperature for 1 hour in a humidified chamber. The solution was decanted and cells washed three times in PBS, 5 min per wash. The FITC-conjugated secondary antibody in 1% BSA was then added and incubated for 1 hr at room temperature in the dark. The cover slip was later washed three times with PBS for 5 min each time in the dark and mounted with a drop of mounting medium. The coverslips were sealed with nail polish to prevent drying and stored in dark at −20 °C until imaging.

#### Angiogenesis Assay

The tube formation ability of the endothelial-like pMSCs was assessed using a tube formation assay. Matrigel basement membrane matrix (10 μl, BD Bioscience, North Ryde, NSW, Australia) was used to coat the inner well of an angiogenesis u-slide (IBIDI, Hallam, VIC, Australia). The Matrigel was allowed to polymerize for 45 minutes at 37 °C in a 5% CO_2_ incubator after which a 50 μl cell suspension containing either 2 × 10^5^ pMSCs/ml in EGMV media with or without BPs (0.5 μM ZA, 1 μM ALN, 2 μM CLO) or HUVECs in EGMV were added. After 6 hours of incubation at 37 °C, cell growth and tube formation were imaged following fluorescent staining with Calcein AM (6.25 μg/ml). Wimasis WimTube (Wimasis GmbH Munich, Germany) image analysis was used to quantify the tube length, number of branching points, total area covered and the number of loops formed in BP treated cells compared with that of the control untreated cells.

### Endothelial cell marker Gene expression

#### RNA isolation, reverse transcription and RT-PCR

Total RNA was isolated from pMSCs after 1, 5 and 10 days of culture in BP supplemented endothelial differentiation media using Trizol reagent (Sigma-Aldrich, Castle Hill, NSW, Australia). Total RNA was quantified by Nanodrop (Thermo Scientific, Rockford, IL, USA) before 1 μg was transcribed into cDNA (Promega, Madison, WI, USA) as per the manufacturer’s instructions. Real time PCR analysis (KAPA SYBR^^®^^ FAST qPCR Kit, Kapa Biosystems, Boston, MA, USA) of stem and endothelial marker gene expression (CD73, CD105, PECAM-1, VEGFR1, VEGFR2, von Willebrand Factor and CD34) was subsequently performed using specific primers (Integrated DNA Technologies, San Diego, CA, USA) on a Real Time PCR system (IQ5, BioRad Laboratories, Hercules, CA, USA). The PCR cycling conditions used were 3 min at 95 °C for enzyme activation, 40 cycles of 3 s at 95 °C, 20 s at 52–58 °C (depending upon the primer annealing temperatures) and 2 s at 72 °C. The specificity of the primers (Table 1) was assessed by melt curve analysis. Each expression analysis was carried out using two individual samples in triplicate and the threshold cycle values averaged. Relative normalized gene expression was calculated using the ΔΔCt method. The results were normalized against the housekeeping gene glyceraldehyde-3-phosphate dehydrogenase (GAPDH).

### Statistical Analysis

All data were expressed as mean ± standard deviation. Comparison between groups were analysed by analysis of variance (ANOVA, post hoc test: Tukey). The software SPSS 17.0 for windows was used for calculations and p < 0.05 was considered to be statistically significant.

## Additional Information

**How to cite this article**: Sharma, D. *et al.* The effect of bisphosphonates on the endothelial differentiation of mesenchymal stem cells. *Sci. Rep.* **6**, 20580; doi: 10.1038/srep20580 (2016).

## Figures and Tables

**Table 1:** Human primers and their sequences used for the stem cell and endothelial gene expression analysis.

Gene	Reference sequence	Primers	Product length
CD73	NM_002526.3	FP: 5′-AGTCCACTGGAGAGTTCCTGCA-3′	132
		RP: 5′-TGAGAGGGTCATAACTGGGCAC-3′	
CD105	NM_000118.3	FP: 5′-CGGTGGTCAATATCCTGTCGAG-3′	109
		RP: 5′-AGGAAGTGTGGGCTGAGGTAGA-3′	
vWF	NM_000552.3	FP: 5′-CCTTGAATCCCAGTGACCCTGA-3′	157
		RP: 5′-GGTTCCGAGATGTCCTCCACAT-3′	
PECAM 1	NM_000442.4	FP: 5′-AAGTGGAGTCCAGCCGCATATC-3′	133
		RP: 5′-ATGGAGCAGGACAGGTTCAGTC-3′	
VE-Cadherin	NM_001795.3	FP: 5′-GAAGCCTCTGATTGGCACAGTG-3′	112
		RP: 5′-TTTTGTGACTCGGAAGAACTGGC-3′	
VEGF-R1	NM_001160030.1	FP: 5′-CCTGCAAGATTCAGGCACCTATG-3′	118
		RP: 5′-GTTTCGCAGGAGGTATGGTGCT-3′	
VEGF-R2	NM_002253.2	FP: 5′-GGAACCTCACTATCCGCAGAGT-3′	132
		RP: 5′-CCAAGTTCGTCTTTTCCTGGGC-3′	
CD34	NM_001025109.1	FP: 5′-CCTCAGTGTCTACTGCTGGTCT-3′	144
		RP: 5′-GGAATAGCTCTGGTGGCTTGCA-3′	
